# In conversation with Dr. María Fernanda Gómez

**DOI:** 10.1111/tpj.71208

**Published:** 2026-11-10

**Authors:** Luis de Luna Valdez

**Affiliations:** ^1^ University of California Riverside Riverside California USA

**Figure:**
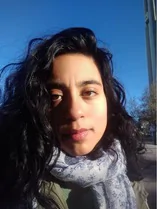
@mfegoz

Originally from Cuernavaca, Mexico, Dr. María Fernanda Gómez turned an encounter with the resurrection plant, *Selaginella lepidophylla*, into a career in plant biology. She studied biology at Morelos State Autonomous University (UAEM) and pursued graduate training at the Institute of Biotechnology of the National Autonomous University of Mexico (UNAM). She later joined Professor Katie Dehesh's laboratory at the University of California, Riverside, where she expanded her expertise in plant biochemistry and metabolomics. Dr. Gómez now leads the Metabolomics Unit at the Centre for Research in Agricultural Genomics (CRAG) in Barcelona, Spain.

In this interview, she discusses how her life experiences shaped her scientific interests and her transition from laboratory research to leading a metabolomics facility. She discusses her recent paper in *The Plant Journal* investigating the role of the Conserved Oligomeric Golgi complex in Golgi organization and plant stress responses.

Dr. Gómez also reflects on navigating international academic environments as a Mexican woman of color and a mother. She offers her personal perspective on scientific development, setting professional boundaries and the importance of belonging within the plant science community. Our conversation explores these matters, offering a space to reflect on the expectations of academic life, the relationships that support professional growth, and the importance of maintaining a sense of self through change.

1. Can you tell us about you and your upbringing?

2. How did you become interested in science and biology?

3. What has your career path been like? What is your current role and how did you land it?

4. Was there a particular mentor, teacher, or moment that inspired and motivated you?

5. What were the biggest barriers you faced early on (cultural transition, finances, confidence), and what helped you push through as a woman of color in academia?

6. Looking back, what do you wish someone had told you about the hidden curriculum of academia (how to find mentors, choose labs, publish, or navigate setbacks)?

7. In general terms, what are your research interests? How did that lead to your latest paper on TPJ (https://doi.org/10.1111/tpj.71077)?

In our recent paper in The Plant Journal, we investigated the role of the Conserved Oligomeric Golgi (COG) complex in Golgi organization and plant stress responses. We found that COG7 is essential for maintaining normal Golgi size and organization. Interestingly, although COG7 has a unique role, we also identified partial functional compensation by COG3 and COG6 (Figure 1), something that had not been reported in earlier works. Importantly, some of these responses can be genetically separated, suggesting that Golgi dysfunction influences different cellular processes through distinct mechanisms. For me, this project was particularly meaningful because it brought me back to my original research interests in membrane trafficking, while allowing me to apply the broader perspective on plant stress signaling and organelle function that I developed at UCR.

**Figure 1:**
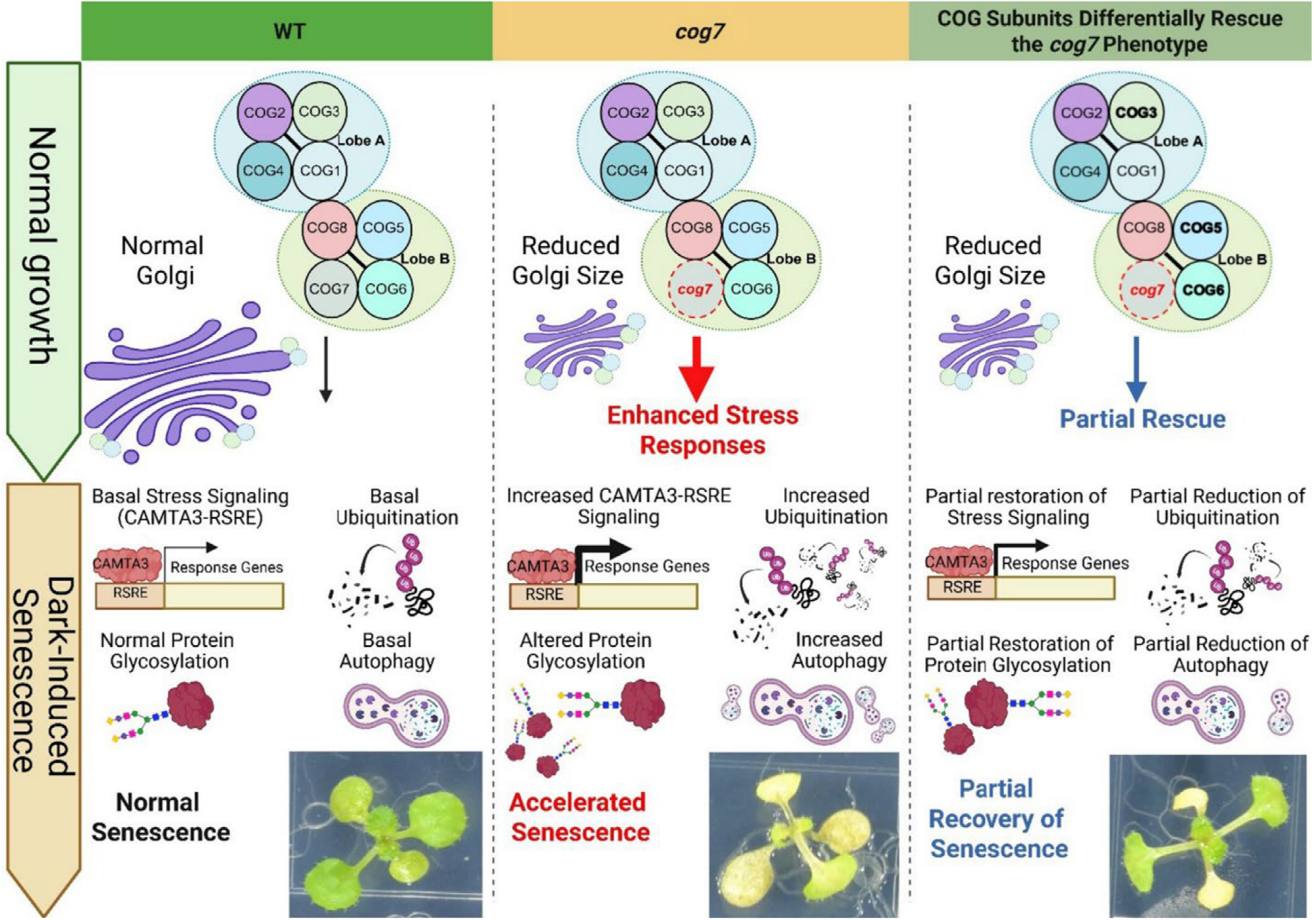
The role of the Conserved Oligomeric Golgi (COG) complex in Golgi organization and plant stress responses.

8. What are the skills you learned through your research that now enable you to perform in your current position?

I would say that the most important skills are related to metabolomics, particularly LC–MS‐based analytical methods and data analysis. I have hands‐on experience working with different mass spectrometry platforms, including high‐resolution mass spectrometry and triple quadrupole instruments. This allowed me to develop skills in sample preparation, chromatographic separation, method optimization, instrument troubleshooting, and quantitative analysis. I also have experience analyzing complex metabolomic datasets, particularly hormone profiles and other stress‐related metabolites. I learned how to approach metabolite identification, evaluate analytical results, and connect changes in metabolite abundance with the biological questions behind each experiment. One of the most valuable aspects of my experience was working on projects involving different research groups. This helped me understand that metabolomics is not simply about generating data, but also about selecting appropriate analytical approaches, recognizing the limitations of each method, and helping researchers interpret their results.

I think all these experiences have prepared me well for my current position leading the Metabolomics Unit at CRAG. Although I am now taking on additional responsibilities like management and organization, I can build on the technical and analytical knowledge I have to support other researchers. I also believe that my background in plant biology and biochemistry is an important advantage because it allows me to understand the biological context of the samples and communicate more effectively with researchers about their experimental goals.

9. What is your current role in the North American Arabidopsis Steering Committee (NAASC)? How do you use this platform to support and empower the plant biology community?

I am currently involved in two NAASC committees: the Early Career Scholars Committee and the Inclusivity Scholars Committee. Through these groups, we organize activities aimed at bringing together plant scientists across North America, sharing knowledge, creating opportunities for professional development, and strengthening our scientific community. Some of the activities we have organized include webinars such as ‘Finding Your Way into Scientific Publishing: From Peer Review to Editorial Roles’, which aimed to help researchers understand how scientific publishing works, the different roles involved in the editorial process, and how they can become involved in peer review and editorial activities. We also organized ‘LGBTQ+ Voices in Science & Community’, an event focused on increasing visibility and recognizing the diversity of people who contribute to science. Scientists come from many different backgrounds and identities, and I believe that acknowledging and celebrating this diversity is one of the simplest ways we can help build a strong scientific community.

We are also preparing an article titled ‘Research in Our Hands’, which aims to highlight the research and personal experiences of people working with plant science across North America. The idea is to showcase not only science but the people behind it, their backgrounds, and perspectives. What motivates me the most about these activities is the opportunity to reach scientists beyond my circle and create a stronger sense of community and belonging. Being part of NAASC has been a very exciting and rewarding experience. It has also given me the opportunity to understand how scientific organizations operate behind the scenes and appreciate the effort involved in organizing initiatives for the community. More importantly, it has reinforced my belief that these organizations are essential not only for advancing science but also for bringing together and supporting the people who make science possible.

10. Do you have any advice for young plant scientists, especially those who are new to research or navigating big transitions?

My advice would be to not be afraid of exploring new opportunities, even when they take you outside your comfort zone. Sometimes, stepping into something unfamiliar can open doors you never imagined. We cannot plan or predict every single step, so it is important to remain open to new possibilities. Looking into different programs, training, and research communities can help expand your options. I would also say to listen to other scientists and learn from their experiences. Everyone has a different journey, and their perspective can teach valuable lessons. I would also encourage them to trust their abilities but be open to learning and recognizing the areas where they can continue improving. You don't need to know everything from the beginning, and growth happens through effort and dedication.

Finally, I think it is important not to forget about your personal life and well‐being. Finding balance helps us maintain the energy and clarity of mind that scientific work requires. Of course, everyone has their own way of working, and finding that balance is also part of the learning process. If I could summarize my advice, I would say: *Be patient with yourself, take every opportunity to learn, take care of yourself, and strive to be the kind of scientist your community needs*.

## Data Availability

Data sharing not applicable to this article as no datasets were generated or analysed during the current study.

